# A Case of Poisoning with Oxalic Acid

**Published:** 1889-09

**Authors:** W. Koehler


### A CASE OF POISONING WITH OXALIC ACID.

(Reported by Dr. W. Kœhler and translated by S. L.)

B. K., of Berlin, nineteen years old, bought sixpence worth Oxalic acid, dissolved it in a glass of water and drank. Shortly before his suicidal attempt he had lunched on three glasses beer, bread and cheese and coffee. In five minutes vomiturition and copious vomiting, burning pains in throat and abdomen. Dr. Kœhler was immediately summoned, and washed the stomach out several times—the water and the vomited matter were bloody. Half an hour later grave collapse; pulse small, irregular, slow (48 per minute); respiration superficial (10–12 to the minute); skin covered with cold, clammy sweat; livid face, features sunken; pupils dilated. Patient opens his eyes when loudly spoken to and then falls back into his apathetic state, constant desire to vomit, but only brings up some mucus—no blood. Hypodermics of Camphor, hot coffee, brandy. About eight P. M., two hours later, he complains of great thirst, of burning and stitching in abdomen, and then collapse returned in the most threatening manner, with cyanosis; livid fingers and toes, dyspnœa, sensory disturbances in toes and tips of fingers. Excitantia relieved again, the pulse regained some volume, great restlessness set in, with tonic and clonic spasms in upper and lower extremities. Especially strong are the patellar reflexes on both sides, as a slight touch causes long-continued, crampy motions on both sides. Achilles tendon reflex and periost. reflex increased. When merely touching the left malleolus ext. the tendon of the left musc. tibialis antic. protrudes. Triceps tendon reflex and periost reflex of the epiphyses of the forearm also increased on both sides; sensorium dull; patient reacts only on loud calls; pupils dilated. Nine P. M. the spasms diminish in strength and frequency; sensorium more free; knows that he is in the hospital; severe pains in throat when talking or swallowing; pulse better; patient sleeps naturally, early interrupted by weak clonic spasms in upper and lower extremities. Ten P. M. abdomen soft—he wants to urinate, but passes only a small quantity (100 ccm) of yellow, somewhat murky urine of acid reaction. Sensorium free and replies freely, though with a hoarse voice and some exertion. Half-past eleven, examination shows perfect anæsthesia of the tips of fingers and toes, of the anterior surface of the hands, and the plantar surface of the feet. While the prick of a pin is rightly localized in the upper arm and thigh, a deep prick is necessary on the leg to cause a dull pain; sensibility on other parts of the body normal, pulse 80, temp. 38.2; pains in the left lumbar region and in both legs; feet and hand as if asleep; great thirst, burning in mouth; sleeps only little during the night.

February 27th, 2d day.—He urinates spontaneously a slightly bloody urine (150 ccm), of acid reaction, leaving a strong sediment; sp. gr. 1016; rich in albumen; epithelia and epithelial cylinders; red and white blood corpuscles; coloring matter of the blood; no sugar; small quantities of Oxalic acid crystals (form of letter-envelopes), amorphous, and in the form of so-called dumb-bells. After standing thirty-six hours the microscope shows copious, whitish, shining crystals of Oxalic acid and of Oxalate of Lime in many forms; insoluble in Acetic, soluble in Muriatic acid.

In the evening pains in renal and vesical region, a crawling sensation over the whole body, especially in both lower extremities. Several times during the day painful spasms in the muscles of the left calf; no appetite, tendency to vomit; profuse perspiration on hands and feet; discharges 300 ccm dark jumentous urine; tongue heavily coated, pulse 84, temperature 38.6.

February 28th, 3d day.—Urine 400, copious albumen and cylinders; sensory disturbances continue; no spasms; dullness in head, patient feels as if had been drunk. Urine 600, no crystals; normal temperature; feet and hands dry.

February 29th, 4th day.—Urine 900, less albumen and cylinders; feels more like himself; sensory and reflex manifestations disappear; slight gastric disturbances still, but the next morning he felt well enough to be discharged.

The lunch taken before the suicidal attempt, and the immediate washing out of the stomach probably saved the patient's life.

